# Transcranial magnetic stimulation (TMS) in a child diagnosed with hypothalamic-pituitary tumour: a case report

**DOI:** 10.1192/j.eurpsy.2023.358

**Published:** 2023-07-19

**Authors:** A. Moleon, M. Martín-Bejarano, J. Narbona, T. Rosa, I. Pérez, M. García-Ferriol, R. Perea, J. M. Oropesa, T. Javier

**Affiliations:** ^1^ Hospital Universitario Virgen del Rocío; ^2^Instituto Andaluz de Salud Cerebral, Sevilla; ^3^Hospital Universitario 12 de Octubre, Madrid; ^4^Universidad de Cádiz, Cádiz; ^5^Instituto Andaluz de Salud Cerebral, Huelva; ^6^Hospital Universitario Virgen Macarena, Sevilla; ^7^ Hospital Juan Ramon Jimenez; ^8^Universidad de Huelva, Huelva, Spain

## Abstract

**Introduction:**

Central nervous system (CNS) tumours are the most common type of solid tumour in the paediatric population. Although advances in treatment have improved survival rates, there is a substantial body of literature documenting the potential long-term effects such as psychological, neurocognitive and health-related sequelae experienced by survivors of paediatric brain tumours. TMS is a non-invasive brain stimulation technique that uses electrical stimuli applied to the cranial surface to restore neuronal connections damaged because of CNS disruption (Burke et. al., 2019).

**Objectives:**

To test the efficacy of TMS in a patient diagnosed with a CNS tumour who reported pain and suffered severe cognitive-behavioural alterations refractory to other pharmacological treatments.

**Methods:**

*Case Presentation.* A 12-year-old boy diagnosed with a hypothalamic-pituitary tumour at the age of 9, having received surgical treatment, radiotherapy and chemotherapy. He suffered loss of vision, cognitive-behavioural and emotional sequelae, and pain, for which he received various pharmacological treatments without benefit. *Treatment.* The patient underwent a total of 25 sessions where each session took 20 minutes to complete for 3 sessions per week. TMS intervention consisted of 1200 inhibitory magnetic pulses with a frequency of 1hz on right DLPFC at an intensity of 110% of resting motor threshold. Stimulations were carried out using a Magventure MagPro X100 equipment with a double-cone coil. The clinical assessment included The Silhouettes Fatigue Scale (PHQ-9), Pain Catastrophizing Scale (*PCS*) and Numerical Rating Scale (NRS), verbal subtests of the Weschler Intelligence Scale for Children (WISC-V), Patient Health Questionnaire (PHQ-9) and the Sleep Disturbance Scale for Children, SDSC

**Results:**

In the post-treatment clinical interview with the family, qualitative changes included a decrease in subjective complaints of pain and fatigue. The family reported that the child stopped sleeping tied up after the intervention and a significant change in slowness was observed, which was accompanied by a higher level of awareness and consequently a slight improvement at the behavioural level, which at the present time does allow for psychological intervention. The psychometric results were clinically improved for psychomotor activity, sleep, emotional alterations, and all cognitive domains.

**Conclusions:**

25 sessions of TMS in the right DLPFC could show beneficial effects on pain, fatigue, cognition, health and sleep variables in patients with drug-resistant sequelae derived from CNS tumours. Longitudinal studies with larger sample sizes are needed to determine whether the effects observed after TMS intervention in paediatric patients with CNS diseases are significant.

**Disclosure of Interest:**

None Declared

